# Relative importance of potential risk factors for dementia in patients with hypertension

**DOI:** 10.1371/journal.pone.0281532

**Published:** 2023-03-15

**Authors:** Mi-Hyang Jung, Kwang-Il Kim, Jun Hyeok Lee, Ki-Chul Sung

**Affiliations:** 1 Department of Internal Medicine, Division of Cardiology, Seoul St. Mary’s Hospital, College of Medicine, The Catholic University of Korea, Seoul, Republic of Korea; 2 Catholic Research Institute for Intractable Cardiovascular Disease, College of Medicine, The Catholic University of Korea, Seoul, Republic of Korea; 3 Department of Internal Medicine, Seoul National University Bundang Hospital, Seoul National University College of Medicine, Seongnam, Republic of Korea; 4 Department of Biostatistics, Wonju College of Medicine, Yonsei University, Wonju, Republic of Korea; 5 Department of Internal Medicine, Division of Cardiology, Kangbuk Samsung Hospital, Sungkyunkwan University School of Medicine, Seoul, Republic of Korea; Ehime University Graduate School of Medicine, JAPAN

## Abstract

Patients with hypertension are at higher risk for dementia than the general population. We sought to understand the relative importance of various risk factors in the development of dementia among patients with hypertension. This population-based cohort study used data from the Korean National Insurance Service database. Using the Cox proportional hazard model, R^2^ values for each potential risk factor were calculated to test the relative importance of risk factors for the development of dementia. Eligible individuals were adults 40 to 79 years of age with hypertension and without a history of stroke and dementia between 2007 and 2009. A total of 650,476 individuals (mean age, 60 ± 11 years) with hypertension were included in the analyses. During a mean follow-up of 9.5 years (±2.8 years), 57,112 cases of dementia were observed. The three strongest predictors of dementia were age, comorbidity burden (assessed using the Charlson Comorbidity Index), and female sex (R^2^ values, 0.0504, 0.0023, and 0.0022, respectively). The next strongest risk factors were physical inactivity, smoking, alcohol consumption, and obesity (R^2^ values, 0.00070, 0.00024, 0.00021, and 0.00020, respectively). Across all age groups, physical inactivity was an important risk factor for dementia occurrence. In summary, controlling and preventing comorbidities are of utmost importance to prevent dementia in patients with hypertension. More efforts should be taken to encourage physical activity among patients with hypertension across all age groups. Furthermore, smoking cessation, avoiding and limiting alcohol consumption, and maintaining an appropriate body weight are urged to prevent dementia.

## Introduction

Dementia is a clinical syndrome that leads to the deterioration of cognitive function beyond the usual consequences of aging, ultimately limiting the ability to perform daily activities. Currently, more than 55 million individuals globally have dementia, and this number is expected to increase to 78 million by 2030 [1]. Dementia is a major cause of disability, dependency, and death for older adults [2]. In Korea, dementia is also expected to become an important health issue since Korea is one of the most rapidly aging countries worldwide. The prevalence of dementia in Korea is estimated to be 10.3% among those over 65 years in 2020 and 15.9% by 2050 (3.02 million people) [3].

Patients with hypertension are at higher risk for incident dementia than the general population [4]. This is partly attributable to the fact that both hypertension and dementia are the effects of aging, and they share common risk factors [5]. Furthermore, recent studies have shown that mid-life hypertension is associated with incident dementia [6–9]. Intriguingly, one study showed that the pattern of association between blood pressure (BP) and dementia development was different depending on whether antihypertensive drugs were taken or not [9]. Because of the considerable disease burden and lack of effective therapy for dementia, identifying and prioritizing the risk factors for dementia are essential components of the primary prevention of hypertension, which is still a growing chronic disease burden in Korea that affected 10.1 million people as of 2019 [10]. A previous study reported that approximately one-third of Alzheimer’s disease cases might be attributable to potentially modifiable risk factors, such as physical inactivity, smoking, hypertension, obesity, and diabetes [9]. Although age is usually regarded as the most powerful but non-modifiable risk factor, dementia is neither an inevitable consequence of aging nor a disease exclusive to older adults [2]. The onset of dementia at a young age (younger than 65 years) has recently received more attention as another important but underappreciated disease entity [11,12].

Therefore, we sought to understand the relative importance of various risk factors for dementia for patients with hypertension, who are at higher risk for developing incident dementia. Additionally, we investigated the age-specific relevance of these risk factors. We anticipate that the data acquired during this study could serve as basic epidemiologic data for establishing effective public health policies and applying individualized (subgroup-specific) preventive strategies.

## Materials and methods

### Data source

We extracted data from the National Health Insurance Service (NHIS) National Health examinee database. The NHIS is the only insurance provider in Korea, covering 97% of the population. National health screening (blood pressure and anthropometric measurements, questionnaires about health status, laboratory tests, and chest X-ray) was conducted every 2 years for the Korean population 40 years or older. The NHIS National Health examinee database includes information about demographic characteristics, diagnoses based on the International Classification of Diseases, tenth revision (ICD-10) codes, admissions, prescriptions, health screening data, and death. Detailed information about the NHIS database can be found elsewhere [13,14].

### Study population

Using the NHIS National Health examinee cohort, we identified 1,406,333 individuals (age 40–79 years) with hypertension between 2007 and 2009 (index period). Among these, we excluded 83,135 subjects with a history of stroke or dementia at baseline using the 2002 to 2006 database information. We further excluded those who did not undergo a health screening examination during the index period (n = 672,667) and those with missing data (n = 55). Ultimately, 650,476 individuals comprised the final study population and were included in the analysis. The study protocol was approved by the institutional review board of Kangbuk Samsung Hospital (KBSMC 2021-08-022). The review board waived the requirement for informed consent because anonymized and de-identified information was provided to the researchers under the strict confidentiality protocol of the NHIS.

### Data collection

Trained staff members measured BP at least twice, with the patient in the sitting position after ≥5 min of rest. The body mass index (BMI) was calculated as weight divided by the height squared (kg/m^2^). Information regarding physical activity, smoking history, and alcohol consumption was collected using self-report questionnaires. Blood samples (fasting glucose and lipid profiles) were collected after ≥8 h of fasting. Income level was classified according to an individual’s insurance premium status. The Charlson comorbidity index was calculated to explore the role of comorbidities in the development of dementia [15].

Hypertension was defined as the presence of a claim for the prescription of antihypertensive medications with relevant ICD-10 codes for hypertension (I10-I13 and I15). Diabetes was defined as the prescription of antidiabetic medications with ICD-10 codes for diabetes (E08-E13). Furthermore, stroke was defined by ICD-10 codes for stroke (I60-64) with hospitalization for ≥2 days.

### Study outcomes and follow-up

The primary study outcome was a new diagnosis of dementia. Dementia was defined when the following two conditions were simultaneously satisfied: corresponding ICD-10 codes for dementia (F00 or G30 for Alzheimer’s disease; F01 for vascular dementia; and F02, F03, or G31 for other dementia) and the prescription of anti-dementia medications (rivastigmine, galantamine, memantine, or donepezil). The accuracy of the codes for dementia according to the NHIS data has been previously tested using the Mini Mental State Examination; the positive predictive value was 94.7% [16]. The study population was followed-up until the development of dementia, death, or the end of the study (December 2017).

### Statistical analysis

To explore the relative importance of the risk factors in the development of dementia, we calculated the estimated explained relative risk (R^2^) using the Cox proportional hazards model. R^2^ values provide an estimate of how important each risk factor is for predicting the outcome [17]. The estimated explained relative risk model has been previously tested during large cohort studies [17–19]. Potential risk factors are as follows: age (40–59, 60–69, and 70–79 years); sex; comorbidity burden (Charlson comorbidity index ≥75^th^ percentile of the study population or <75^th^ percentile of the study); physical inactivity (≥1 time/week or never); smoking habit (current smoker or others); alcohol consumption (≥10 g/day or <10 g/day); obesity (BMI ≥27.5 kg/m^2^ or <27.5 kg/m^2^); use of statins; elevated fasting glucose level (≥140 mg/dL or <140 mg/dL); BP control status (systolic BP ≥130 mmHg or <130 mmHg); hypertension duration (≥5 years or <5 years); elevated total cholesterol level (≥200 mg/dL or <200 mg/dL); and low-income level (lowest quartile or others). Among many drugs, we sought to identify the role of statin use on the development of dementia for two reasons. First, statin use might reflect the atherosclerotic burden of vessels, which might affect vascular dementia. Second, apolipoprotein E (APOE) gene polymorphism might be related to Alzheimer’s disease and lipid profile [20]. Thus, we identified the role of statin in the occurrence of dementia of Alzheimer’s or vascular-type dementia. The analyses were applied to the overall hypertensive population and age subgroups. All analyses were performed using SAS version 9.4 (SAS Institute, Inc., Cary, NC, USA).

## Results

### Baseline characteristics

A total of 650,476 individuals with hypertension (mean age, 60.2 ± 11.2 years; men, 48.9%) were included in the analyses. The mean systolic BP and diastolic BP were 134.1 ± 17.0 and 82.1 ± 11.0 mmHg, respectively; 50.9% of the population had hypertension for a duration ≥5 years. A total of 23.1% of the population had diabetes, and 51.5%, 28.6%, and 19.9% of the population had BMI <25, 25–27.4, and ≥27.5 kg/m^2^, respectively. The other baseline characteristics are listed in the S1 Table. During a mean of 9.5 ± 2.8 years of follow-up, 57,112 cases of dementia developed.

### Relative importance of risk factors for dementia in the overall population

**Fig 1:**
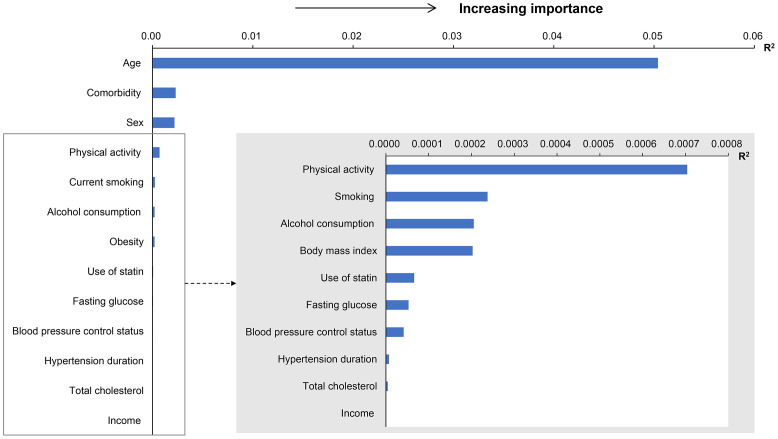
The relative importance of risk factors for the development of dementia among patients with hypertension.

Age showed the highest R^2^ value (0.0504) for dementia (Fig 1). After age, comorbidity burden and female sex (R^2^ values, 0.0023 and 0.0022, respectively) had the next highest R^2^ values for dementia. These were followed by physical inactivity, smoking, and alcohol consumption, which are lifestyle factors (R^2^ values, 0.00070, 0.00024, and 0.00021, respectively). Obesity, statin use, elevated fasting glucose levels, and BP control status were followed as other risk factors for dementia.

### Subgroup analyses by age

**Fig 2:**
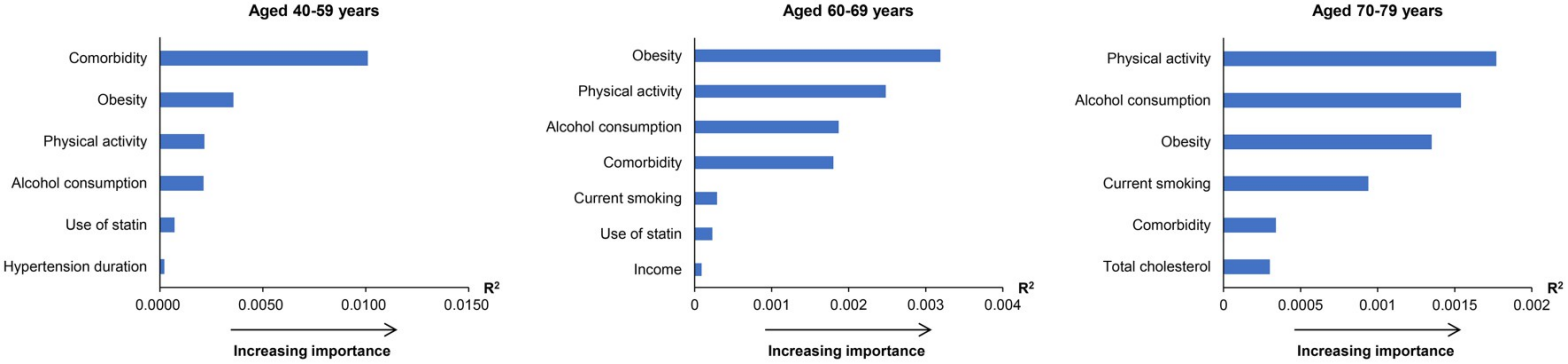
Age-specific relative importance of risk factors for the development of dementia among patients with hypertension: Subgroup analyses by age.

Across all three age groups, physical inactivity was included among the three most important risk factors, with the highest R^2^ value for dementia (Fig 2). The three most important risk factors for middle-aged adults (age 40–59 years) were comorbidity, obesity, and physical inactivity. The three most important risk factors for older adults (age 60–69 years) were obesity, physical inactivity, and alcohol consumption. Finally, for the oldest adults (age 70–79 years), the three most important risk factors were physical inactivity, alcohol consumption, and obesity.

## Discussion

In this nationwide cohort of 650,476 individuals with hypertension, we found that age, female sex, and comorbidity burden are fundamental risk factors for the development of dementia. Physical inactivity, smoking, alcohol consumption, and obesity are also important risk factors for dementia. Among these lifestyle-related factors, physical inactivity is the most powerful risk factor for dementia. Our results revealed possible targets for prevention and their relative importance for patients with hypertension who are at higher risk for dementia. Furthermore, to the best of our knowledge, our study is the first to determine the relative importance of various risk factors in the occurrence of dementia among patients with treated hypertension.

The prevalence of dementia is expected to increase in parallel with the rapidly aging population. As demonstrated during the current study, age is the most important risk factor for dementia and is regarded as an unmodifiable risk factor. However, it should be pointed out that aging is not an inevitable consequence of age [21]. Recently, abundant data suggested that biological age is more important than chronological age. It is crucial to focus more attention on lifestyle and environmental risk factors that could affect biological age, such as comorbidity burden, physical activity, current smoking, obesity (BMI ≥27.5 kg/m^2^), and alcohol consumption (≥10 g/day), which were all high-ranking risk factors for dementia during the current analyses. Among various lifestyle factors, physical inactivity was the most powerful risk factor for dementia in this study. Evidence supports that aerobic exercise may attenuate cognitive impairment and reduce dementia risk [22–24]. Exercise and regular physical activity may benefit cognitive function by regulating amyloid ß turnover, reducing inflammation, promoting neurogenesis through neurotrophin production, and improving cerebral blood flow [22–24]. It is important to note that the salutary effects of regular physical activity and exercise apply to all ages, including older adults. Several randomized clinical trials have demonstrated better cortical connectivity and larger hippocampal volume in older adults after aerobic exercise [25,26]. Moreover, the benefit of physical activity could be applied to cognitive function and vascular health, which might be particularly essential in patients with hypertension. The brain of a hypertensive patient shows increased microvascular rarefaction, decreased cerebral flow, and disrupted brain-blood barrier integrity. However, physical activity could ameliorate or reverse those changes by increasing vascular endothelial growth factor or enhancing brain-derived natriuretic factor (a molecule related to neuronal survival and synapse formation) [27]. A meta-analysis involving 19 prospective studies found that for older adults (mean age, 74 years), current smoking was associated with a 79% increased risk of Alzheimer’s disease, 78% increased risk of vascular dementia, and 27% increased risk of any type of dementia compared to never smoking [28]. This study clearly showed that individuals (including older adults) should quit smoking to prevent dementia. In terms of alcohol consumption, most data support the risk of heavy alcohol consumption for patients with dementia. However, there is some controversy regarding the amount of alcohol allowed [29–31]. In the current study, alcohol consumption of any amount (≥10 g/day [1 unit/day]) was a risk factor, particularly for older hypertensive patients (age 60 years or older). Previous meta-analyses demonstrated that even low amounts of alcohol consumption are associated with the risk of hypertension for Asian men [32]. Avoiding and limiting alcohol consumption, especially for Asian individuals, might improve cognitive health. However, additional studies are warranted. Regarding obesity, there is a discrepancy regarding appropriate BMI levels, often referred to as the obesity paradox (lower incidence of dementia among obese individuals or lower mortality among obese individuals with dementia) [33–35]. To accurately assess this paradox, separate evaluations before and after the onset of dementia are warranted. When the scope is limited to risk factors for dementia development, obesity appears to be a risk factor for dementia, especially for middle-aged and older adults. Some studies have shown that obesity prevents the development of dementia among the oldest adults [34]. However, several studies have demonstrated that obesity increases the risk of future dementia in the long term (>10 years from baseline) and has a paradoxical association in the short term [35]. Weight loss often precedes the onset of dementia by 10 years (preclinical stage of dementia). This may be a confounding factor, especially for the oldest adults with a shorter follow-up period. In the current study, obesity (BMI ≥27.5 kg/m^2^) was a significant risk factor for dementia development across all age groups, which could serve as evidence supporting weight control. However, this needs to be cautiously interpreted because we did not evaluate the risk of being underweight. In the current analyses, BP control status or hypertension duration was not a high-ranking risk factor, except in younger adults. This might partly be attributable to the fact that our cohort comprised treated hypertensive patients who were on antihypertensive medications, and their BP was relatively well controlled (the prevalence of individuals with systolic BP < 130 mmHg was almost half). A prior study has shown that the relationship between BP and the risk of dementia might differ based on taking antihypertensive medications [7]. Other BP-related parameters (e.g., BP variability or white-coat phenomenon) might be more important in incident dementia [36].

Age-specific differences in the priority of risk factors also need to be discussed. Generally, the overall trend (i.e., physical inactivity and obesity as important risk factors) was similar across all age groups. One of the intriguing findings in the current study was the relative importance of comorbidity or hypertension duration in the younger adults treated with hypertension. Dementia in older adults (age 75 years or greater) is mainly because of Alzheimer’s disease, which originates from the long-term accumulation of amyloid deposits. Epigenetic factors, such as physical activity or alcohol consumption, can play a more important role in modifying the genetics of Alzheimer’s disease rather than hypertension duration in older adults [37]. Conversely, dementia in young and middle-aged adults (40–74 years) is mainly because of genetic factors (early-onset Alzheimer’s disease, frontotemporal dementia) or vascular factors (vascular dementia). Indeed, comorbidity burden and hypertension duration, which might be associated with vascular dementia, were identified as important risk factors in this study in younger adults aged 40–59. Similar to our results, Jung et al. previously reported that increased BP was associated with an increased risk of dementia in younger individuals (< 70 years old) but not in an older population (>70 years) [8]. Further studies are needed to clarify the age-specific etiologies in developing dementia.

Several strengths of the current study are as follows. First, the potential risk factors and their relative importance for dementia were evaluated among a large population of hypertensive patients treated with antihypertensive medication. To the best of our knowledge, this is the first study that explicitly prioritized and determined the relative importance of various risk factors for the occurrence of dementia, an important public health issue.

This study also had some limitations that should be discussed. First, causal inferences might have been limited because our study was based on a retrospective cohort study. Second, we lack information on a comparator group of an age and sex-matched general population without hypertension. Third, there were no data on other important risk factors for dementia, such as family history, genetic factors, and medication histories other than statin use, which might have resulted in the extremely low R_2_ values. Fourth, we did not evaluate the influence of each component of comorbidity on the occurrence of dementia, which is also a relevant study theme that warrants additional studies. Fifth, we defined dementias, which incorporate Alzheimer’s disease, vascular dementia, and other forms of dementia. Therefore, this study could not differentiate the importance of various risk factors for each type of dementia. In this context, our results should be cautiously interpreted in the context of Korean patients with hypertension generated based on the data collected herein.

## Conclusions

Age and sex are fundamental risk factors for the development of dementia in patients with hypertension. Furthermore, comorbidity burden, physical inactivity, smoking, alcohol consumption, and obesity are other important risk factors for dementia. Therefore, controlling and preventing comorbidities are of utmost importance in preventing dementia in patients with hypertension. More efforts should be taken to encourage physical activity among patients with hypertension across all age groups. Furthermore, smoking cessation, avoidance of and limiting alcohol consumption, and maintaining an appropriate body weight are urged for the primary prevention of dementia.

## Supporting information

S1 Table Baseline characteristics of the study population based on age. (DOCX)
