# Supplementary material for: Relative importance of potential risk factors for dementia in patients with hypertension
Source: PLoS One. 2023 Mar 15;18(3):e0281532. doi: 10.1371/journal.pone.0281532 (PMC10016665; doi:10.1371/journal.pone.0281532)
Supplement: S1 Table — (DOCX) [file pone.0281532.s001.docx]

**S1 Table. Baseline characteristics of the study population based on age.**

| **Variables** | **Total** | **Aged 40-59** | **Aged 60-69** | **Aged 70-79** |
| --- | --- | --- | --- | --- |
| Participants, n (%) | 650,476 (100) | 296,026 (45.5) | 208,019 (32.0) | 146,431 (22.5) |
| Sex, n (%) |  |  |  |  |
| Men | 318,191 (100) | 165,513 (55.9) | 94,402 (45.4) | 58,276 (39.8) |
| Women | 332,285 (100) | 130,513 (44.1) | 113,617 (54.6) | 88,155 (60.2) |
| Age, years | 60.2 ± 11.2 | 50.2 ± 6.7 | 64.3 ± 2.9 | 74.6 ± 4.3 |
| SBP, mmHg | 134.1 ± 17.0 | 133.1 ± 16.7 | 134.5 ± 16.8 | 135.6 ± 17.6 |
| DBP, mmHg | 82.1 ± 11.0 | 83.5 ± 11.2 | 81.4 ± 10.5 | 80.3 ± 10.8 |
| SBP categories, mmHg |  |  |  |  |
| <130 | 322,616 (49.6) | 154,008 (52.0) | 100,258 (48.2) | 68,324 (46.7) |
| ≥130 | 327,678 (50.4) | 141,933 (48.0) | 107,686 (51.8) | 78,030 (53.3) |
| HTN duration, years | 4.8 ± 2.8 | 4.4 ± 2.8 | 5.1 ± 2.8 | 5.2 ± 2.8 |
| HTN duration categories, years |  |  |  |  |
| <5 | 319,153 (49.1) | 161,646 (54.6) | 93,634 (45.0) | 63,835 (43.6) |
| ≥5 | 331,378 (50.9) | 134,380 (45.4) | 114,385 (55.0) | 82,596 (56.4) |
| Fasting glucose, mg/dL | 105.9 ± 32.1 | 105.3 ± 32.6 | 106.5 ± 31.6 | 106.1 ± 31.8 |
| Fasting glucose categories, mg/dL |  |  |  |  |
| <140 | 594,207 (91.4) | 271,194 (91.6) | 189,261 (91.0) | 133,704 (91.4) |
| ≥140 | 56,005 (8.6) | 24,756 (8.4) | 18,641 (9.0) | 12,601 (8.6) |
| Known diabetes | 150,107 (23.1) | 54,893 (18.5) | 55,424 (26.6)) | 39,772 (27.2) |
| DM duration, years | 6.5 ± 2.0 | 6.1 ± 2.0 | 6.7 ± 1.9 | 6.8 ± 1.8 |
| DM duration categories, years |  |  |  |  |
| <5 | 561,873 (86.4) | 264,386 (89.3) | 174,461 (83.9) | 122,985 (84.0) |
| ≥5 | 88,658 (13.6) | 31,640 (10.7) | 33,558 (16.1) | 23,446 (16.0) |
| Body mass index, kg/m^2^ | 25.0 ± 3.2 | 25.4 ± 3.2 | 25.0 ± 3.0 | 24.2 ± 3.2 |
| <25 | 334,670 (51.5) | 139,953 (47.3) | 106,046 (51.0) | 88,639 (60.6) |
| 25-27.4 | 185,901 (28.6) | 87,989 (29.7) | 61,755 (29.7) | 36,143 (24.7) |
| ≥27.5 | 129,647 (19.9) | 68,010 (23.0) | 40,141 (19.3) | 21,487 (14.7) |
| Smoking status |  |  |  |  |
| Non-smoker | 431,052 (71.9) | 183,093 (64.4) | 130,609 (76.0) | 117,305 (81.9) |
| Ex-smoker | 69,363 (11.6) | 38,091 (13.4) | 18,570 (10.8) | 12,698 (8.9) |
| Current smoker | 98,992 (16.5) | 63,109 (22.2) | 22,714 (13.2) | 13,167 (9.2) |
| Alcohol frequency |  |  |  |  |
| None | 377,224 (62.4) | 145,359 (50.7) | 118,389 (68.3) | 113,436 (78.8) |
| 2/month-2/week | 164,209 (27.2) | 107,894 (37.6) | 37,626 (21.7) | 18,681 (13.0) |
| 3-7/week | 62,759 (10.4) | 33,662 (11.7) | 17,238 (10.0) | 11,856 (8.2) |
| Physical activity |  |  |  |  |
| None | 313,027 (51.8) | 131,397 (45.8) | 89,480 (51.7) | 92,122 (64.0) |
| Yes | 290,900 (48.2) | 155,354 (54.2) | 83,769 (48.4) | 51,755 (36.0) |
| Income status, quartile |  |  |  |  |
| Lower 30% (low) | 140,116 (22.0) | 65,899 (22.7) | 47,972 (23.6) | 26,245 (18.2) |
| Middle 40% | 219,060 (34.3) | 107,831 (37.1) | 70,323 (34.6) | 40,906 (28.4) |
| Upper 30% | 278,872 (43.7) | 117,121 (40.3) | 84,870 (41.8) | 76,881 (53.4) |
| Use of aspirin | 266,964 (41.0) | 101,525 (34.3) | 95,410 (45.9) | 70,001 (47.8) |
| Use of statin | 163,234 (25.1) | 70,636 (23.9) | 58,428 (28.1) | 34,156 (23.3) |
| Total cholesterol, mg/dL | 198.5 ± 40.1 | 199.2 ± 39.5 | 198.3 ± 40.2 | 197.5 ± 41.3 |
| Total cholesterol categories, mg/dL |  |  |  |  |
| <200 | 356,620 (54.8) | 160,559 (54.3) | 114,175 (54.9) | 81,857 (55.9) |
| ≥200 | 293,638 (45.2) | 135,403 (45.8) | 93,744 (45.1) | 64,465 (44.1) |
| Charlson Comorbidity Index | 3.85 ± 1.90 | 2.54 ± 1.32 | 4.21 ± 1.45 | 5.57 ± 1.69 |

DBP, diastolic blood pressure; DM, diabetes mellitus; HTN, hypertension; SBP, systolic blood pressure
